# A Mitosis Block Links Active Cell Cycle with Human Epidermal Differentiation and Results in Endoreplication

**DOI:** 10.1371/journal.pone.0015701

**Published:** 2010-12-20

**Authors:** Jennifer Zanet, Ana Freije, María Ruiz, Vincent Coulon, J. Ramón Sanz, Jean Chiesa, Alberto Gandarillas

**Affiliations:** 1 Cell Cycle, Stem Cell Decision and Cancer Laboratory, Institute for Training and Research of the Fundación Marqués de Valdecilla (IFMAV-FMDV), Molecular Biology Department of Universidad de Cantabria (UC), Santander, Spain; 2 Laboratoire de Dermatologie Moléculaire, UPRES EA3754, Institut Universitaire de Recherche Clinique, UMI/INSERM, Montpellier, France; 3 Inserm ADR Languedoc - Roussillon, Montpellier, France; 4 Unité Fonctionnelle de Cytogénétique Anténatale et Oncologique, CHU Nîmes, Nîmes, France; 5 Servicio de Cirugía Plástica, Hospital Universitario Marqués de Valdecilla (HUMV), Santander, Spain; Center for Regenerative Therapies Dresden, Germany

## Abstract

How human self-renewal tissues co-ordinate proliferation with differentiation is unclear. Human epidermis undergoes continuous cell growth and differentiation and is permanently exposed to mutagenic hazard. Keratinocytes are thought to arrest cell growth and cell cycle prior to terminal differentiation. However, a growing body of evidence does not satisfy this model. For instance, it does not explain how skin maintains tissue structure in hyperproliferative benign lesions. We have developed and applied novel cell cycle techniques to human skin *in situ* and determined the dynamics of key cell cycle regulators of DNA replication or mitosis, such as cyclins E, A and B, or members of the anaphase promoting complex pathway: cdc14A, Ndc80/Hec1 and Aurora kinase B. The results show that actively cycling keratinocytes initiate terminal differentiation, arrest in mitosis, continue DNA replication in a special G2/M state, and become polyploid by mitotic slippage. They unambiguously demonstrate that cell cycle progression coexists with terminal differentiation, thus explaining how differentiating cells increase in size. Epidermal differentiating cells arrest in mitosis and a genotoxic-induced mitosis block rapidly pushes epidermal basal cells into differentiation and polyploidy. These observations unravel a novel mitosis-differentiation link that provides new insight into skin homeostasis and cancer. It might constitute a self-defence mechanism against oncogenic alterations such as Myc deregulation.

## Introduction

Correctly co-ordinating cell growth and differentiation is essential to morphogenesis and adult tissue homeostasis. Human epidermis is a self-renewal stratified epithelium that is highly exposed to mutagenic hazard and frequently affected by hyperproliferative lesions. Within epidermis, proliferation is confined to the basal layer and differentiation takes place as keratinocytes migrate through the suprabasal layers [1], [2]. The balance between proliferation and differentiation must be tightly controlled and must lie on the relationship between the cell cycle [3] and terminal differentiation. This relationship remains enigmatic.

Epidermal keratinocytes undergo two transitions regarding the proliferative state as they progress along the differentiation programme: i) when daughters of the stem cells in the basal layer enter a clonal expansion phase of rapid proliferation and become what has been defined as transit amplifying cells (TAC); and ii) when these cells cease to proliferate and undergo terminal differentiation. Interestingly, cells that are in the rapid proliferation phase are committed to differentiate by unknown mechanisms after four or five rounds of cell division [1]. As keratinocytes leave the basal layer and initiate terminal differentiation, their intermediate filament cytoskeleton changes from proliferative keratins 5 and 14 to the post-mitotic keratins 1 and 10 [4], [5], [6]. Traditionally, proliferative keratinocytes have been assumed to exit the cell cycle into G0 (cell growth arrest) before they initiate terminal differentiation. This model however does not explain a growing body of evidence: i) keratinocytes grow in size during differentiation [7], [8], [9]; ii) some unexplained mitotic figures or thymidine-incorporating cells have been reported in the peribasal layer [10], [11], [12], [13], [14]; iii) inhibiting keratinocytes entry in cell cycle did not provoke terminal differentiation in a variety of studies [15]; for instance, over-expression of the cell cycle inhibitor p21CIP rather inhibited differentiation [16], [17]; iv) a temporal gap between keratinocyte cell cycle arrest and terminal differentiation has not been observed [13], [15], [18]; v) primary keratinocytes can differentiate terminally from any phase of the cell cycle and differentiating cells are not predominantly in G0/G1 but rather they accumulate in G2/M [19]; vi) constitutive activation of the cell cycle inducer, proto-oncogene Myc in human keratinocytes or mouse epidermis stimulates differentiation [20], [21], [22]; vii) finally and not less important, benign hyperproliferative lesions of skin consistently associate epidermal hyperplasia with hyperkeratosis (thickening of the differentiated strata), as it occurs in a variety of transgenic mouse lines over-expressing cell cycle molecules in epidermis including E2F [23], cyclin D1 [24], [25], MDM2 [26], cdk4 [27] or cdk2 [28]. Therefore, a good amount of evidence suggests that epidermal differentiation does not require cell cycle arrest.

We have previously shown that primary differentiating keratinocytes continue DNA synthesis in the absence of cell division and become polyploid in culture [15], a phenomenon referred to as endoreplication. Interestingly, keratinocyte endoreplication and size are stimulated by Myc activation [15] and inhibited when Myc is inactivated in mouse epidermis [29]. Linking differentiation to cell cycle progression might contribute to maintain tissue structure, even upon molecular alterations that otherwise would be oncogenic. It was therefore important to determine whether this mitosis-defective cell cycle has physiological significance and it occurs in human differentiating epidermis.

We have studied the epidermal relationship between cell cycle and differentiation and applied innovating techniques to assess DNA replication and DNA content in human epidermis *in situ*. We report the spatial pattern of expression of key cell cycle and DNA replication molecules and a functional link between a mitosis block and differentiation. We show that actively cycling keratinocytes of normal skin initiate terminal differentiation without suppressing DNA replication and become polyploid. This phenomenon appears widespread within epidermis and ubiquitous within human skin, since we have detected it in skin from various adult body sites. Interestingly, although epidermal endoreplication has been disregarded in humans, it is evolutionary conserved with plants and invertebrates [30], [31], [32], [33]. These findings provide novel clues into the understanding of human epidermal homeostasis and carcinogenesis.

## Results

### Differentiating human epidermis contains polyploid cells and nuclei

**Figure 1:**
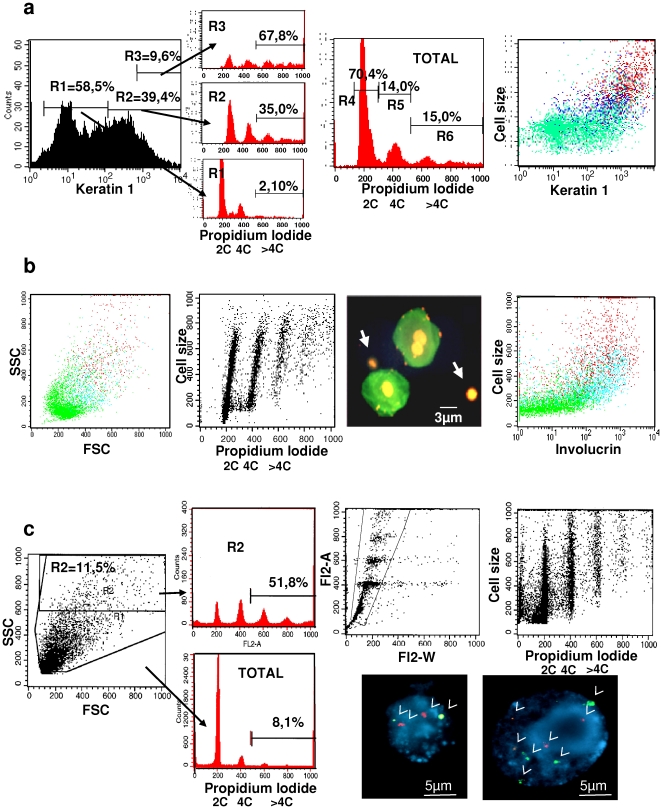
Polyploid cells and nuclei in epidermal cell suspensions. (**a–c**): representative flow-cytometry analysis of freshly isolated keratinocytes (a–b) or nuclei (c) from human epidermis. Side (SSC) and Forward (FSC) Scatter reflect cell size and complexity. FL2 reflects propidium iodide (PI, DNA content: 2C, 4C, >4C or polyploidy). Coloured dots are cells in the different phases of the cell cycle (G1: green; S/G2/M: blue; polyploidy: red). Numbers show percent of cells within each region for each histogram. a: Keratinocytes from foreskin were double labelled for postmitotic keratin K1 and DNA. Cells were sorted on basis of low K1 (R1), high (R2) and very high K1 (R3) and the DNA content of the different subpopulations analysed. The dot Plot represents K1 versus cell size expression. Histograms represent DNA content of all cells (TOTAL) or of K1 negative cells (R1), according to negative antibody b: Keratinocytes from breast skin were fixed and double stained for involucrin and DNA. Dot plots represent SSC and FSC (left), PI versus cell size (middle) or involucrin expression versus cell size (right), as indicated. The photograph shows keratinocytes stained with involucrin (green) and PI (red); note the smaller nuclei negative for involucrin (arrows). c: Freshly isolated nuclei from human breast epidermis dot plots represent light Scatter (left), FL2-Width versus FL2-Area (first right: typical analyses to exclude aggregates) or nuclear size (light scatter) versus DNA content (second-right). Red histograms represent DNA content of all nuclei (TOTAL) or nuclei with higher size and complexity (R2), on basis of the light scattering. Photographs show FISH on diploid (left) or polyploid (right) nuclei stained with probes for centromere 20 (red; 2 dots on the left, 4 dots on the right) or Her2Neu locus (green; two dots on the left, four dots on the right; also note the difference in nuclear size). The data are representative of at least three independent experiments.

We isolated keratinocytes freshly from human epidermis and analysed their DNA content by propidium iodide staining and flow-cytometry (Fig. 1a). Differentiating keratinocytes become bigger and more complex (cellular components) and can thus be identified on the basis of the increase in their forward scatter (FSC) and side scatter (SSC) parameters [15], [34]. However, only part of the differentiating population can be obtained upon a trypsin treatment of epidermis, since late differentiating keratinocytes are strongly attached. Nonetheless, we have consistently found a significant proportion of polyploid cells in epidermal cell suspensions from different body sites, which was consistently restricted to the differentiating compartment (red spots; Fig. 1a–b). The DNA content of keratinocytes correlated with cell size (Fig. 1a,b). As larger and more differentiated populations were analysed the G1 peak of the cell cycle decreased whereas the G2/M and polyploid fractions increased (Fig. 1a,b). This suggests that eventually most differentiating cells become polyploid. By staining for two differentiation markers, involucrin or keratin 1, we confirmed that keratinocytes with the largest size and the highest DNA content, mostly polyploid cells, were differentiated (Fig. 1a,b). Around 35% of keratin 1-positive cells and 25% of involucrin-positive cells were polyploid.

Known animal endoreplicating systems contain both multinucleate cells and polyploid nuclei (e.g., [32], [35], [36], [37]). Cells with two nuclei can be isolated from epidermis (Fig. 1a,b). To determine whether the epidermis also contains polyploid nuclei, we isolated epidermal nuclei by pepsin treatment of isolated keratinocytes or whole epidermis (Fig. 1c). By doing this we also ruled out a potential contribution of aggregates into the polyploid fraction in the whole-cell analyses. The presence of single polyploid nuclei was confirmed by standard FL2 Area/Width analyses of the DNA staining (FL2-A and FL2-W in Fig. 1c). We obtained a significant proportion of polyploid nuclei with discrete 6C and 8C peaks, again displaying a correlation between size and DNA content (Fig. 1c). We also detected chromosome amplifications in isolated nuclei by Fluorescence *In Situ* Hybridisation (FISH) with probes for specific chromosome centromeres (arrows; photos in Fig. 1c).

**Figure 2:**
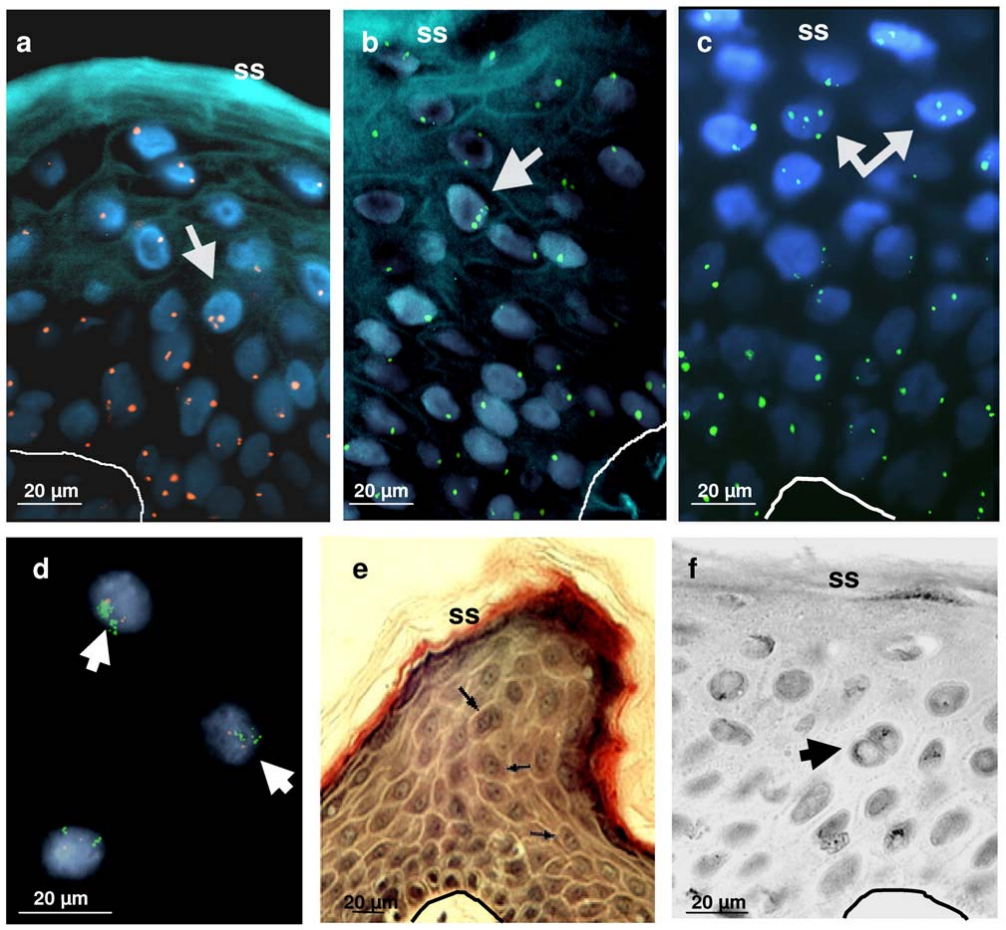
Chromosome amplifications and binucleate cells in suprabasal human epidermis. **a**,**b**,**c**: Fluorescence *In Situ* Hybridisation (FISH) for centromeres of chromosome 8 (a, red), 18 (b, green) or 20 (c, green) on human skin sections from healthy breast (a,b) or foreskin (c). Nuclei stained with the Dapi compound in blue. Similar results were obtained for chromosomes 8,9,18 and 20. **d**: isolated nuclei from breast epidermis hybridised for centromere of chromosome 9 (red), Her2Neu locus (green) and DNA (blue). Arrows indicate nuclei with cromatide amplifications. **e**, **f**: hematoxylin/eosin (e) or giemsa (f) staining of human skin sections from scalp (e; slightly cross-cut) or breast (f). Arrows indicate binucleated cells. The white or black line represents the basement membrane that links the epidermis with the underlying dermis. SS: skin surface.

Since isolating cells or nuclei from differentiating layers of epidermis is technically difficult, we aimed to identify polyploid nuclei *in situ*. To this end we applied the FISH technique onto human skin sections. Hybridising for chromosome-specific centromeres revealed the presence of frequent chromosome amplifications in normal suprabasal epidermis of different body sites (Fig. 2a–c). We obtained similar results with all centromeric probes utilised (chromosomes 8, 9, 18, 20, see Mat. and Meth.), further confirming the existence of polyploid nuclei and suggesting that the whole genome is amplified in those nuclei. Nuclei with three or four spots were consistently found in suprabasal differentiating cells and never in the basal, proliferative layer, nor in the first suprabasal layer (Fig. 2a–c). However, fluorescent hybridisation within epidermal sections is limited due to hard access into the tissue and to the sectioning of hybridised nuclei in two pieces. As a result many centromeres are lost and in fact, many nuclei or nuclear fragments contained one or non spot in our studies. For these reasons, the FISH technique is not suitable for quatitation of polyploidy in situ. Nonetheless, we never observed more than four spots per nuclei, suggesting that at some point of the differentiation programme chromatids are no longer able to separate. Indeed, some of the isolated nuclei from epidermis contained chromatid multiplications (Fig. 2d). Cells with two nuclei were found in suprabasal layers of skin of different body sites (Fig. 2e,f). Binucleate cells are typical of endoreplicating systems (e.g.,[35]).

**Figure 3:**
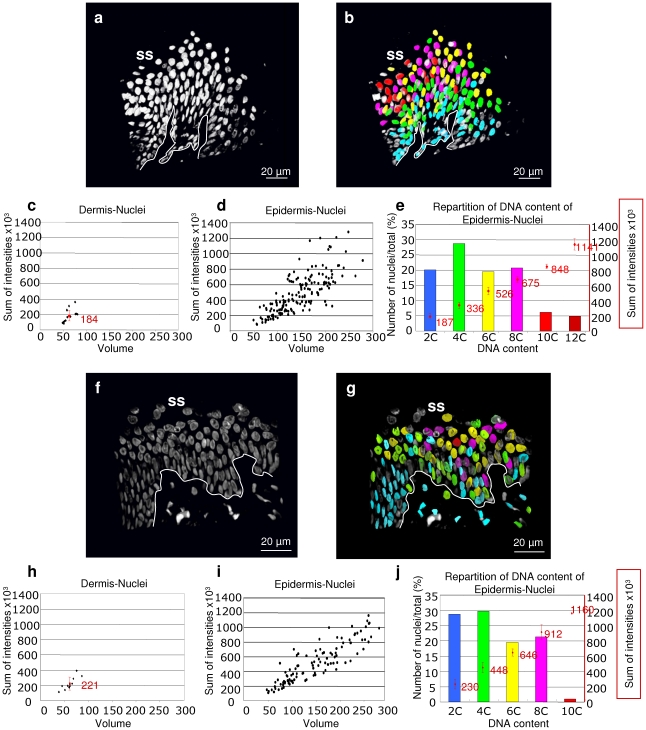
*In situ* quantitation of nuclear DNA of human epidermis *in situ*. **a**,**b**,**f**,**g**: 3D reconstruction of epidermis of foreskin (a,b) or scalp (f,g) stained for DNA with the Dapi compound, showing the nuclei in grey scale (a,f) or in rainbow colours corresponding to the estimated DNA content (b,g). Grey nuclei in b,g were incomplete and therefore disregarded. **c**,**d**,**h**,**i**: dot plots showing volume versus sum of fluorescence intensity values of nuclei in the dermis as control (c, h) or in the epidermis (d, i). **e**,**j**: approximate distribution of nuclei according to their estimated DNA content (left axis). Values in red are the means of the sum of intensities of the corresponding group of nuclei (right axis). Bars are standard deviations. White lines: basement membrane. SS: skin surface. Two different fields per individual were analysed.

Since flow-cytometry analyses of isolated keratinocytes or FISH might underestimate the proportion of polyploid cells and nuclei and cannot show their location, we developed a method to quantitate nuclear DNA *in situ*. To this aim we labelled ‘thick’ skin microsections (25 µm) with the DAPI compound to stain for nuclear DNA and performed three-dimensional (3D) tissue-reconstruction. Nuclear DNA content and volume were then measured according to the amount of fluorescence of the 3D objects (Fig. 3a,f; see Mat. and Meth.). A link between DNA quantitation and the spatial location was kept. Dermal nuclei were analysed as a diploid control (Fig. 3c,h). As shown in Fig. 3, most nuclei within epidermis had an estimated DNA content higher than 2C and spread out along the volume/intensity histogram (Fig. 3d,i). A significant proportion of nuclei had a 4C estimated DNA content and a significant proportion had more than 4C, with maximal ploidies reaching 12C (Fig. 3e,j). As suggested by the flow-cytometry analyses, nuclei were increasingly polyploid as they were higher-up within the epithelium (Fig 3b,g). No perfect correlation was found between DNA content and specific layers. However, there was a correlation between nuclear volume and DNA content, and these correlated in turn with differentiation (Fig. 3 d,i,b,g). 4C nuclei (around 30%; Fig. 3e,j) were found mainly in peribasal layers, but were also present in most suprabasal layers. Conversely, very few diploid nuclei were detected in superficial layers (Fig. 3b,g). Altogether, this suggests that most differentiating keratinocytes eventually undergo endoreplication and only a small proportion remain in G0/G1. It is technically very complex to accurately quantitate the proportion of polyploid cells within the epidermis. It must also be noted that differentiating 4N cells should be post-mitotic and therefore they are also polyploid. Our analyses estimated around 30% of 4C nuclei in foreskin or scalp epidermis and 52% of >4c in foreskin or 42% in scalp (Fig. 3e,j). These studies indicate that polyploidisation, rather than sporadic, is a widespread phenomenon in human epidermis.

### The cell cycle stays active in differentiating epidermis

**Figure 4:**
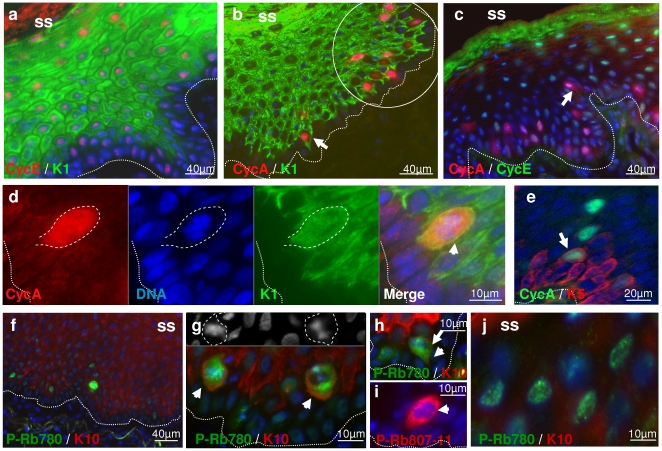
Expression of cell cycle regulators of S phase in normal human epidermis. Expression of cyclin E (Cyc E;a,c), cyclin A (Cyc A;b–e), or phospho-Rb ser780 (P-Rb780;f–h,j) or ser807-811 (P-Rb807-11;i) and differentiation markers keratin 1 (K1;a,b,d), keratin 10 (K10;f–h,j) or keratin 5 (K5;e) as indicated. Colours as indicated. Nuclei stained with Dapi, blue. Circle in b highlight patch of positive cyclin A cells. More details are shown in Fig. S1. **d**,**e**,**h**,**i**: amplified areas of the basal layers. **g**,**j**: amplified area of suprabasal layers. Arrows point at ‘mushroom cells’, arrowheads at mitotic figures. g shows in gray scale the DNA staining of metaphasic figures. Dotted lines indicate the basement membrane; broken lines, metaphasic cells. SS: skin surface. Results are representative of at least four different individuals.

Cyclins control the different transitions of the cell cycle by regulating the activity of cdks (cyclin dependent kinases; reviewed in [38]). We have determined the expression of these cyclins in normal human epidermis (Fig. 4 and Fig. S1). Cyclin E was detected in a heterogeneous fashion in the nuclei of all living layers of epidermis (Fig. 4a and Fig. S1a,b). Strikingly, its expression was moderate and patchy in the basal layer and spread and increased in differentiated layers (Fig. 4a and Fig. S1a,b). Although some basal cells expressed cyclin A, most positive cells were found within the first suprabasal layers (peribasal), coexpressed with the postmitotic terminal differentiation keratins K1 and K10 (Fig. 4b,d and Fig. S1c–e). Often, cells leaving the basal layer that still have a stalk in it but are already in the first suprabasal layer (‘mushroom’ cells; e.g., [12], [39]) were expressing cyclin A (Fig. 4b–e). Positive cells were often found in groups, possibly due to clonal cell cycle activation (Fig. 4b,e and Fig. S1c–e). Some cells in peribasal layers expressed cyclin A but not cyclin E, indicative of G2 phase (Fig. 4c). No cyclin A was detected in more superficial layers that markedly expressed cyclin E (Fig. 4c). The switch from cyclin A to high cyclin E takes place in the epidermal layers where the differentiation marker involucrin begins to be expressed (Fig. S1; Freije et al, unpublished data). Involucrin is a precursor of the cornified envelope and its expression correlates with keratinocyte cell size [7], [8], [9]. The pattern of expression of cyclin E and A in epidermis was confirmed with three and two different antibodies, respectively (see Mat. and Meth.). The most suprabasal cyclin A cells had lost the epidermal proliferative marker keratin 5 antigen (Fig. 4e). Cells co-expressing cyclin E and/or A and keratins 1 or 10 must be in cycle in the absence of cell division (endoreplication), since these cytoskeletal proteins have been shown to impede proliferation in vitro and in vivo [4], [5], [6].

To further study the cell cycle activity in differentiating epidermis, we stained skin with antibodies specific for phosphorylated Rb (P-Rb; ser780; ser807–811; Fig. 4f–j). Phosphorylation of tumour suppressor Rb hallmarks cell cycle activation [40]. Interestingly, positive cells were found mainly in the first suprabasal layers, as observed for cyclin A. ‘Mushroom’ cells that strongly expressed P-Rb contained metaphasic figures (Fig. 4f–i). Moreover suprabasal nuclei retained P-Rb expression (Fig. 4f,j and Fig. S2g). Double labelling for cyclin E and P-Rb showed coexpression in suprabasal nuclei (Fig. S2g). We stained skin further for other molecules involved in cell cycle progression such as PCNA and KI67 (see Fig. S2). They were both expressed in suprabasal layers.

It is worth noting that all cell cycle markers were heterogeneous in suprabasal layers with both positive and negative nuclei. This is consistent with suprabasal nuclei continuing to cycle.

### DNA replication continues in differentiating epidermis

**Figure 5:**
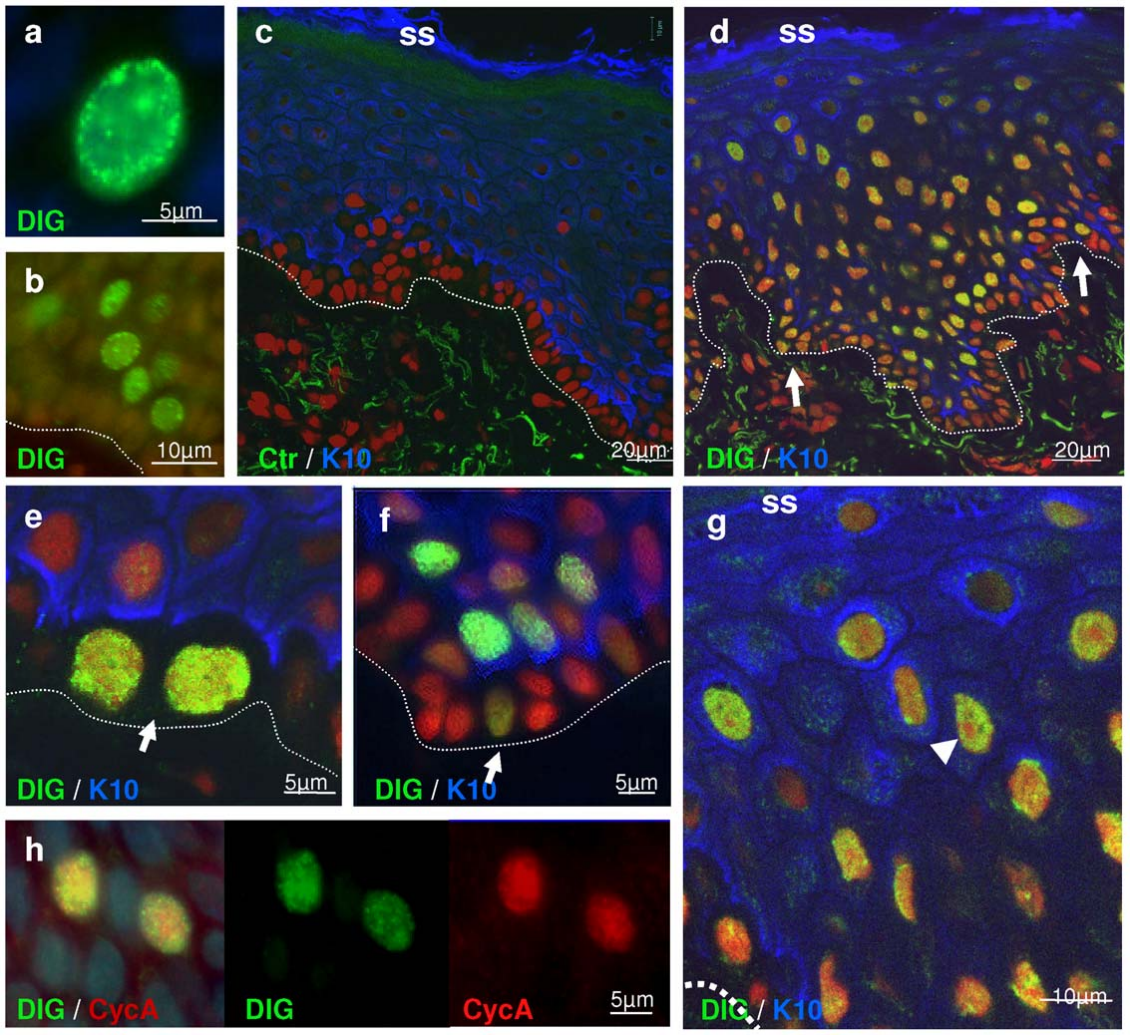
*In situ* DNA replication in human epidermis. Human skin sections were subject to DNA synthesis and stained for digoxigenin (Dig is incorporated in neo-synthesised DNA (see Mat. and Meth.) and keratin 10 (K10) or cyclin A, as indicated. Colours as indicated. Nuclear DNA is stained in blue (Dapi) for conventional microscopy (a,b,h), or in red (propidium iodide) for confocal microscopy (c–g). **c**: negative control where Dig was not added to the reaction (Ctr; see also Fig. S5). **a**,**b**,**h**: sections of neonatal foreskin; **c–g**: adult foreskin. Arrows in d–f point at DNA synthesising basal cells (see also Fig. S3). **g**: amplified area of d. **h**: double labelling for DNA synthesis and cyclin A, a marker of S phase. Arrowhead in **g**: nucleolus. Broken line indicates the basement membrane. SS: skin surface. Note that DNA synthesis spread in more suprabasal layers, generally (not always) showing a sparser pattern. Results are representative of foreskin from more than 5 individuals.

**Figure 6:**
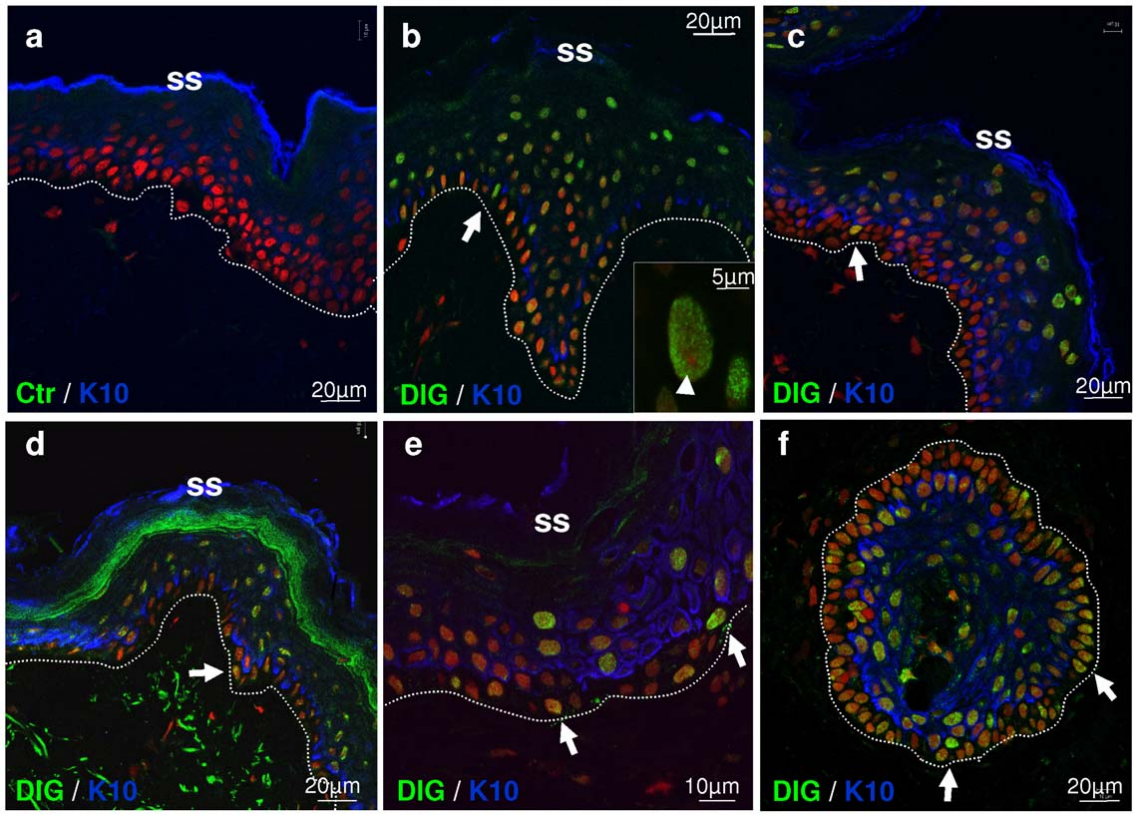
*In situ* DNA replication in human epidermis of various body sites. Digoxigenin incorporation (Dig., green) and keratin 10 (blue). DNA stained in red (propidium iodide) for confocal microscopy. **a**: negative control with Dig, without other nucleotides(Ctr); **a–b**: scalp; **c**: breast; **d**: thigh; **e**,**f**: eyelid. Inset in b shows an amplified suprabasal nucleus. Arrows: DNA synthesising basal cells; arrowhead: nucleolus (b, inset). Dotted line basement membrane. SS: skin surface. Results are representative of at least four different individuals.

Increasing DNA content and the pattern of expression of cell cycle molecules in epidermis strongly suggested that DNA replication remains active in the onset of differentiation. To confirm this, we applied to normal human skin a powerful method to detect DNA replication *in situ* on frozen sections [41]. This method takes advantage of the cell DNA replication machinery that remains active after snap-freezing. DNA synthesis is detected by incorporation of a thymidine analogue (digoxigenin; Dig; see Mat. and Meth.). As shown in Fig. 5, positive nuclei were labelled with a focal pattern typical of DNA replication. Negative controls were performed where either the nucleotide mix or the thymidine analogue Dig was omitted (Figs. 5c and 6a). The pattern of DNA synthesis in epidermis was heterogeneous, with negative or positive nuclei. Interestingly, although some basal cells were undergoing DNA replication, most positive cells were found in suprabasal layers (Fig. 5d–g). Double labelling for DNA replication and keratin 10 showed that most DNA replicating cells expressed the post-mitotic terminal differentiation marker (in blue in confocal microscopy in Fig. 5c–g). Brightest-DNA synthesising cells were often within the basal and peribasal layers, forming patches, suggesting clones of cell cycle activation (Fig. 5b,d,f). These patches were found mainly in the basal layer (e.g., Fig. 5e) or mainly migrating into suprabasal layers (e.g., Fig. 5f). Labelled cells spread out in more suprabasal layers, where DNA synthesis appeared generally sparser (Fig. 5g). The pattern of DNA synthesis was dark in the nucleoli (labelled in red by propidium iodide), as expected for DNA replication. The sparser labelling in nuclei of higher layers might be because of difficult accessibility, or because DNA replication is slower or intermittent in those cells. As expected for DNA replication, some positive cells expressed also cyclin A (Fig. 5h), further indicating that those cells were in S phase. Some cells in peribasal layers expressed cyclin A but were negative for Dig, suggesting they were in G2 (arrows in Fig. S3). Conversely, DNA synthesising nuclei were observed beyond the cells expressing cyclin A (asterisks in Fig. S3), suggesting they were undergoing endoreplication. An artifactual background due to DNA repair appeared in our DNA replication assays with time at 37°C, which we could abolish by using a DNA repair inhibitor (see Data S1, Fig. S4 and Fig. S5). Suprabasal DNA synthesis was further observed in flow-cytometry studies on BrdU incorporation in epidermal sheets *in toto* (Fig. S6).

We next questioned whether epidermal suprabasal replication was a ubiquitous phenomenon through-out the human body and performed the DNA synthesis assay on skin from different sites, including neonatal or adult foreskin, adult breast, eyelid, scalp, abdomen or thigh from different male or female individuals. As summarised in Fig. 6a–f, all body sites studied were positive for suprabasal DNA replication and were labelled with a similar pattern, indicating that differentiation-associated DNA replication is a ubiquitous phenomenon of human skin.

### Suprabasal cells express G2/M markers

**Figure 7:**
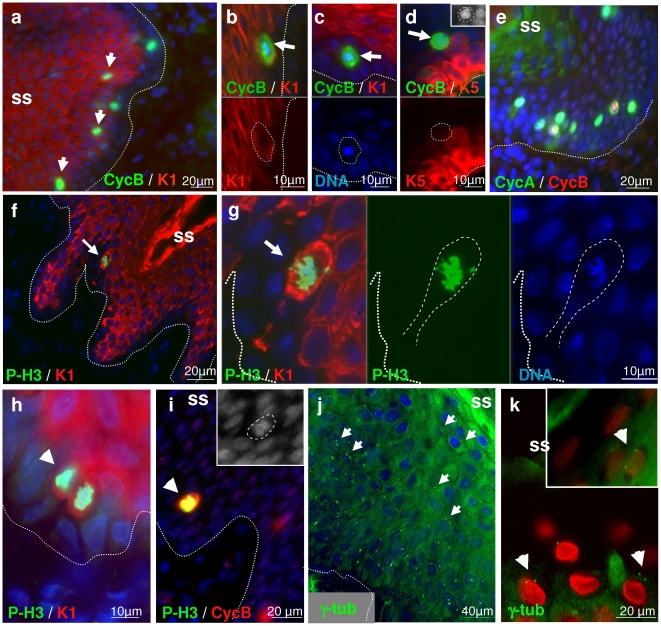
Expression of G2/M markers in human skin. Normal human skin sections were immunostained for Cyclin B (Cyc B;a–e, i), phospho-histone H3 (P-H3;f-i), keratin 1 (a–c,f–h) or keratin 5 (d) or γ-tubuline (γ-tub; j,k; to detect centrosomes; arrows), coloured as indicated. Nuclei were stained with Dapi (a–j, blue) or propidium iodide (confocal microscopy, k, red). Dotted line indicates the basement membrane. SS: skin surface. Arrows: in a–i point at mitotic cells; arrow in f,g at a typical metaphasic “mushroom” cell; in j,k centrosome duplications (j: conventional microscopy; k: confocal microscopy). Images are representative of skin from foreskin or breast of at least three different individuals.

To explore the mitosis block at the initiation of terminal differentiation that results in re-replication, we stained epidermis for mitotic markers. The expression of cylin B takes place in a narrow window at early mitosis and metaphase [3], [38]. We detected some cyclin B1 positive cells in the basal layer of human epidermis (Fig. 7a–c) but they were more frequent within peribasal layers expressing the postmitotic terminal differentiation markers keratins 1 and 10 and no longer positive for the epidermal proliferative marker keratin 5 (Fig. 7d and Table 1). Most epidermal cyclin B1 cells contained condensed, prophasic or metaphasic DNA (arrowheads in Fig. 7). This pattern was confirmed with two different anti-cyclin B antibodies (see Mat. and Meth.). Some cyclin B-positive cells also expressed the S/G2 marker cyclin A. Strong cyclin B expression was found in the layer of cells that initiated the expression of the cornified envelope precursor involucrin (Fig. S1h). As for cyclin B, we detected phospho-H3 histone (P-H3) in the peribasal layer of epidermis, at the front of the first differentiating layer (Fig. 7f–i), often ‘mushroom’ cells. P-H3 is a marker of mitotic chromosome condensation [42]. P-H3 positive cells were also positive for cyclin B1 (Fig. 7i). We did not find strong cyclin B1 or P-H3 cells or metaphase figures beyond the peribasal layers. However, staining for the centrosome marker γ–tubulin revealed frequent centrosome duplications and separation typical of S phase/mitosis [43] all through living epidermis, even in very superficial layers (Fig. 7j,k).

**Table 1:** Distribution of cell cycle markers and DNA replication in human epidermis.

Layer	CycE	CycA	CycB	PRb	PCNA	Ki67	Ctr.	PH3	Repl*
**Superficial**	+++	-	-	++	+	+	2	-	++
**Peribasal**	+/-	+++	++	+++	+++	++	2	++	+++
**Basal**	+	+	+	+	++	+	1	+	+

*Ratio Replication Suprabasal/Basal Cells: Foreskin: 4.81±1.88; other sites: 3.69±1.25.

**Figure 8:**
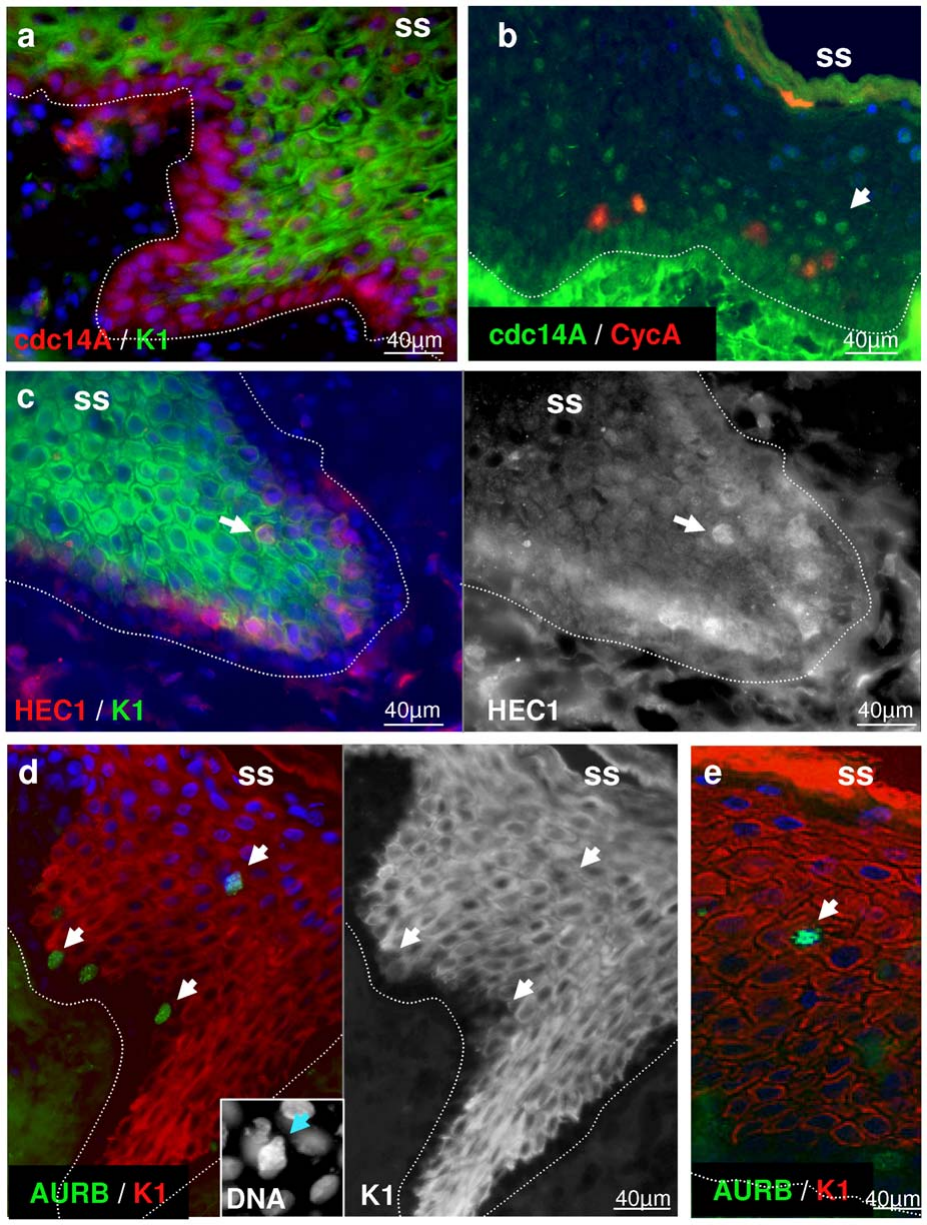
Expression of late Mitosis regulators in human skin. Normal human skin sections were immunostained for cdc14A (a,b), Cyclin A (b), HEC1 (c) or Aurora Kinase B (AURB; d,e), and keratins 1 and 10, colours as indicated. Nuclei were stained with Dapi (blue, a–e). Dotted line indicates the basement membrane. SS: skin surface. Arrows point at suprabasal, differentiating positive cells. Images are representative of foreskin from two different individuals.

The detection of frequent mitotic events in differentiating layers of the epidermis suggested those keratinocytes might be somehow cell cycle arrested in mitosis after entering endoreplication, as it has been described recently for embryo stem cells [44]. To study this possibility further, we determined the pattern of expression of molecules that act in late mitosis, as cells are prone to divide, and are part of the spindle assembly checkpoint. We analysed cdc14A, that regulates centrosome separation and activates the anaphase promoting complex cdh1/APC [45], Ndc80/Hec1 (Highly Expressed in Cancer; [46]) a component of APC and Aurora Kinase B, a key regulator of chromosome segregation [47]. Deregulated cdc14A regulates disrupts chromosome segregation [48]. Up-regulation of cdh1-APC has been shown to destroy mitotic cyclins and trigger endoreplication (e.g.,[49], [50], [51]). As illustrated in Fig. 8, cdc14A is detected in the cytoplasm in patches of basal cells but accumulates in most nuclei of differentiating cells. Double staining showed that as cdc14A accumulates in the nuclei, the detection of cyclin A is lost (Fig. 8a,b). Expression of Hec1 was weak in the basal layer and induced sharply in the peribasal layers (Fig. 8c). Aurora B accumulates in condensed chromosomes of basal and peribasal mitotic cells and strikingly, it is strongly expressed in isolated highly superficial cells of the differentiating layers (Fig. 8d,e).

### A mitosis-differentiation response

**Figure 9:**
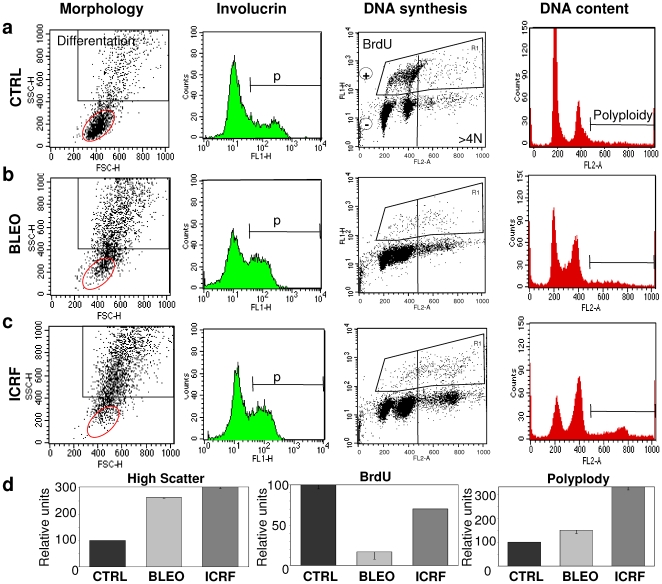
Blocking mitosis by genotoxic insult triggers epidermal differentiation and endoreplication. Keratinocytes freshly isolated from skin treated for 48 h with **a**: DMSO as control; **b**: Bleomycin; **c**: ICRF-193; **d**: statistics of a representative experiment. Morphology, involucrin expression, DNA synthesis (BrdU incorporation) and DNA content were analysed by flow-cytometry as in Fig. 1. SSC: side scatter; FSC: forward scatter; red circle gates for basal cells; black square gates for differentiating cells. The involucrin positive region (p) was determined by a negative isotype antibody control (α-human CD8). Small bars in histograms show s.e.m. of duplicates. Results are representative of experiments with cells from three different individuals.

The pattern of expression of cell cycle regulators within epidermis suggests that the initiation of terminal differentiation associates with a mitosis arrest and slippage [52]. To test whether there is a functional link between both events, we blocked the G2/M transition of freshly isolated keratinocytes from human skin and monitored the effect on terminal differentiation and DNA replication. We performed this by two means: i) use of Bleomycin, that mimics the effect of γ-irradiation by provoking DNA brakes [53] and ii) use of ICRF-193 that inhibits topoisomerase II [54], required for chromosome condensation and segregation. Both treatments block the cell cycle in G2. We then analysed keratinocytes by flow-cytometry as in Fig. 1. A 48 h treatment with either BLEO or ICRF caused in epidermal keratinocytes a dramatic increase of cell size and complexity typical of terminal differentiation, reflected by light scattering and morphology (Fig. 9; “morphology”). The induction of terminal differentiation was further confirmed by the up-regulation of the differentiation marker involucrin (Fig. 9; “Involucrin”). Therefore, both treatments pushed basal cells to differentiate within 48 h. Cells accumulated in G2/M and polyploidy especially in response to ICRF (Fig. 9; ”DNA content”). Yet, keratinocytes were able to bypass the G2/M block and to re-replicate (Fig. 9; “DNA synthesis”). Thus, a significant proportion became polyploid by 48 h (Fig. 9; “DNA content”). These results reveal a functional cause-effect link between a G2/M block and the initiation of terminal differentiation that results in polyploidy and cell size increase.

## Discussion

### Cell cycle regulation and DNA replication

We present novel clues to the coordination between cell growth and differentiation in human epidermis. The diverse techniques that we have utilised are all consistent to show that the cell cycle stays active during differentiation and that a significant proportion of suprabasal cells and nuclei become polyploid. All the parameters of cell cycle progression analysed were positive in suprabasal keratinocytes that expressed post-mitotic keratins K1 and K10. These cytoskeletal proteins have been shown to impede proliferation when ectopically expressed [4], [5], [6]. Therefore, although it seems cautious not to rule out the possibility that some cells migrating into the first suprabasal layer might divide, the results indicate that the cell cycle continues in differentiating keratinocytes in the absence of cell division. We made similar observations studying various body sites including neonatal and adult foreskin and adult breast, scalp, eyelid and thigh, thus suggesting it is a spread out phenomenon within human skin.

**Figure 10:**
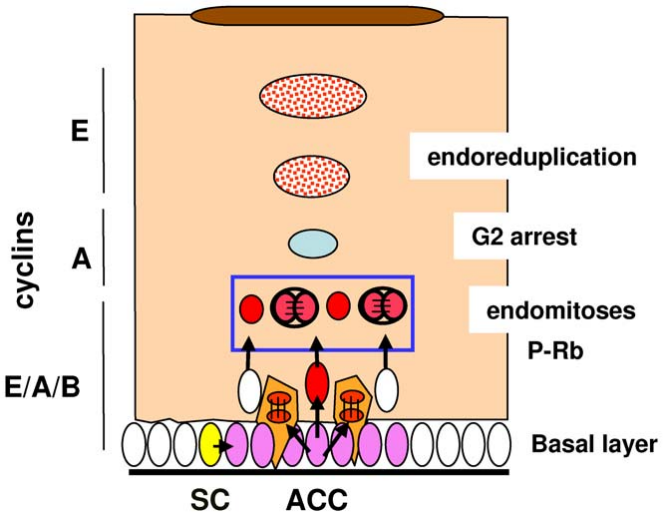
Model for the relationship between cell cycle and differentiation in human epidermis. Stem cells within the basal layer (SC; yellow) by division give rise to actively cycling cells that proliferate rapidly (ACC; pink). ACC lose adherence to the basement membrane, block mitosis, initiate terminal differentiation, migrate into suprabasal layers and continue DNA replication (red). Some of them undergo endomitosis in peribasal layers and become binucleate. Suprabasal keratinocytes lose mitotic cyclins A and B and reinitiate DNA replication in the absence of nuclear division (endoreduplication; punctuated red), the nuclei becoming polyploid. Endomitosis and endoreduplication are different forms of endoreplication.

Both cyclin A and B1 are required for nuclear division [38]. Their pattern of expression in the first suprabasal layers of the epidermis (Table 1), the presence of metaphase figures and the expression of P-H3 in cells that express keratins K1 or K10, indicates that some early differentiating cells undergo nuclear divisions and endomitosis (Fig. 10). This explains the presence of binucleated cells that we have detected in epidermis and previously reported mitotic figures in peribasal layers that remained unexplained [10], [11], [12]. Mitotic figures were not observed in more superficial layers. The absence of cyclin B1 and A and the persistence of cyclin E, PCNA, KI67 and P-Rb in more differentiated layers (Table 1) suggest that suprabasal nuclei undergo endoreduplication: they continue DNA replication and become polyploid once nuclear division takes place no longer (Fig. 10).

The molecular regulation of endoreplication in mammals has been scarcely studied. However, the down-regulation of mitotic cyclins and the accumulation of cyclin E that we have found in human differentiating epidermis and in *in vitro*-induced keratinocyte differentiation (Freije et al, unpublished data), is consistent with studies on endoreplication in human megakaryocytes [55] and trophoblasts [56], in invertebrates [32], [57] and in plants [58], [59]. Interestingly, reported cyclin E knock-out mice are viable, the main alteration observed being impaired endoreplication of trophoblasts and megakaryocytes [60], [61].

The existence of endoreplication in differentiating epidermis was further indicated by the detection of frequent suprabasal chromosome amplifications and polyploid nuclei. In addition, we found centrosome duplication and separation all through suprabasal epidermis and this suggests that suprabasal nuclei stay in a special G2/M state even at very late differentiation. It must be noted here that we found over-expression of cyclin E in suprabasal layers and one of the functions of cyclin E is centrosome duplication [43]. The spread expression of phosphorylated Rb in most suprabasal nuclei further suggests that they do not return to G1. This is in striking agreement with what Mantel et al. (2008) have recently described in embryonic stem cells after mitotic slippage (i.e., bypass the mitotic block to reinitiate DNA replication; [44]). The peribasal over-induction of Ndc80/Hec1, the nuclear accumulation of cdc14A and the presence of strong Aurora B cells in differentiating layers further indicate this notion. We thus propose that differentiating keratinocytes undergo mitotic slippage Physical constrains may prevent differentiating cells from nuclear division and chromosome segregation, thus explaining the increase in nuclear volume and ploidy in suprabasal layers found in the 3D analyses. Indeed, the differentiate cytoskeleton formed by keratins K1 and 10 has been shown to impede proliferation both in vitro and in vivo [4], [5], [6].

Our *in situ* DNA replication studies further showed that suprabasal nuclei undergo DNA replication. The sparser pattern of replication in superficial nuclei might be due to a slower or intermittent suprabasal DNA replication. The estimated time for a keratinocyte to go through all layers of human epidermis is three-four weeks and intermittent but cumulative DNA replication would explain the progressive acquisition of polyploidy. This would agree with the more punctuated nuclear pattern of PCNA in suprabasal layers, possibly due to a lower number of replication origins. The analyses on the expression of γ-H2AX, an early DNA repair marker, confirmed that the pattern of nucleotide incorporation in the conditions of our assays was unrelated to DNA repair. Although it is not technically possible to quantitate accurately the proportion of endoreplicating keratinocytes in epidermis, altogether the results suggest that it is a widespread phenomenon within human skin.

### Sense of epidermal endoreplication

Human epidermal endoreplication might come as a striking finding since cell cycle progression and differentiation are considered mutually exclusive processes. However, some other adult human tissues undergo endocycles during differentiation [35], [36], [37], [62], [63]. Endoreplication might have been disregarded in human epidermis because of the technical difficulties to visualise binucleate cells on skin sections or to isolate late differentiating cells from epidermis and because most polyploid keratinocytes might have a single nucleus. Moreover, although radiolabelled thymidine or BrdU incorporation have been detected in peribasal layers of mouse epidermis, superficial labelling might be elusive due to the diffusion barrier in differentiating layers (see Mat. and Meth.). As for flow-cytometry, most studies disregard cells beyond 4c, or are done in low Ca^+2^ conditions, where differentiating cells detach and are lost into the culture medium (see e.g.,[64]). Nevertheless, a significant proportion of tetraploid and polyploid nuclei was estimated in mouse epidermis by Russian authors by the method of Feulgen for DNA staining [65]; bi-nucleated cells have been reported in the squamous epithelium of normal rodent oral mucosa [66]; nuclei from differentiating rat epidermis were found to retain the capacity to replicate DNA *in vitro* [67]; we have found a proportion of polyploid keratinocytes in normal mouse epidermis by flow-cytometry [29]; and endoreplication is known to be important in plant and invertebrate epidermis [31], [32], [68], [69].

Although not profusely studied in mammals, endoreplication might have important biological roles. Successive rounds of DNA replication in the absence of cell division allow cellular growth to continue after mitotic arrest [32]. In skin, endoreplication explains how keratinocytes progressively increase their size during differentiation. Several reasons might make epidermal endoreplication physiologically important:

- large cells probably improve the resistance of skin to mechanical tension;
- by increasing the cell volumes the total number of cells required for the same body surface diminishes. This in turn diminishes the number of cells generated, the frequence of cell divisions and thus, the probability of gene mutations within the germinative layer caused by environmental hazard such as UV light;
- amplifying gene copy number renders more efficient the production of RNA and proteins. This is important when both cell size increase and a specialised function need to be achieved, as it occurs in epidermal differentiation (see e.g., [59]). Clearly however, the techniques that we have utilised are insufficient to fully determine whether the whole genome of differentiating keratinocytes is amplified or some specific regions contain highly copied amplicons;
- orchestrating homeostasis.

### Orchestrating homeostasis: mitosis and differentiation

A link between active cell cycle and the initiation of terminal differentiation that via a mitosis block gives rise to endoreplication might be important to maintain epidermal homeostasis. Actively cycling keratinocytes were mostly found in the onset of initiation of terminal differentiation (Table 1). These cells were often detaching from the basal layer into the first suprabasal layer (e.g., ‘mushroom cells’; [12], [39]). Therefore, cells that are actively cycling often mitotic appear to initiate terminal differentiation (Fig. 10). These observations would be in agreement with the possibility proposed from studies on mouse epidermis, that asymmetric cell division might help promote keratinocyte upward migration [70]. Suprabasal cells expressed regulators of the spindle assembly checkpoint, APC-related molecules such as cdc14A, HEC1 or Aurora B. Furthermore, when we blocked mitosis of freshly isolated keratinocytes by genotoxic insult, basal cells massively entered differentiation within 48 h. In our studies, nuclei in the peribasal layers were more often in cycle than the nuclei in the basal layer (Table 1). Taken together, the results suggest that the cell cycle is uninhibited in the onset of differentiation. This in turn would imply that only stem cells within the basal layer have their cell cycle inhibited.

Linking initiation of differentiation with an active cell cycle through a mitosis block might constitute a simple mechanism to maintain homeostasis and protect the skin against oncogenic activations. In the event of cell cycle hyper-activation, cells would still undergo terminal differentiation and block proliferation, switch to endoreplication and allow cellular growth to continue. This way, accelerated cell cycle would result in increased differentiation (what overactivation of the proto-oncogene Myc causes in keratinocytes; [20]). Dangerous DNA mutations would no longer have undesirable effects due to the cell division block. In this regard, terminal differentiation would in epidermis counteract apoptosis in other cell systems in order to suppress cell transformation (see [71]). This model would explain why hyperproliferative stimuli in benign skin lesions (hyperplasia) or activation of Myc [20], [21], [22], [72] consistently associates hyperkeratosis (thickening of the differentiating strata) rather than tumorigenesis. In summary, this would explain why alterations stimulating the G1/S transition of the cell cycle in epidermis do not suffice to break down tissue structure and trigger tumorigenesis, unless additional alterations hit the mitosis checkpoints and sustain deregulated proliferation. Now the challenge will be to determine the molecular mechanisms underlying these checkpoints and whether their alteration has responsibility in cancer.

## Materials and Methods

### Histology and immunostaining

#### Ethics Statement

Ethical permission for this study was demanded, approved and obtained from the Ethical Committee for Clinical Research of Cantabria Council, Spain. In all cases, human tissue material to discard after surgery was obtained with written consent presented by clinicians to the patients, the identity was not kept and the material was thus treated completely anonymously.

Normal human skin biopsies were obtained from neonatal or adult circumcisions (foreskin) or adult plastic surgery (breast, scalp, abdomen, eyelid, thigh), frozen, microsectioned, stained by immunofluorescence and analysed under conventional or confocal fluorescent microscopy. The fat and most of the connective tissue was removed using curved scissors. Tissue was embedded in OCT compound (Tissue- Tek, Sakura) and snap-frozen in isopentane (Sigma-Aldrich, Inc.) and frozen in Nitrogen liquid (ThermoShandon). Frozen tissue was microsectioned and 5-10 µm thick frozen sections were collected on glass-slides Superfrost Plus (Thermo Scientific) and air dried for hematoxylin eosin staining. For immunofluorescence staining, they were fixed and permeabilised in methanol (-20°C) or subsequently fixed in 4% formaldehyde and cold methanol, rinsed in PBS and incubated with primary and secondary antibodies diluted in PBS, each for 1 hour at room temperature with PBS washes in between. Sections were stained for DNA with DAPI reagent (Sigma-Aldrich, Inc.) and mounted in Mowiol (Sigma-Aldrich, Inc.) or with the Vectashield + DAPI (Vector Laboratories). For γH2AX staining on pre-fixed skin, the tissue was fixed for 30 min. in 4% formaldehyde. The following primary antibodies were purchased from Santa Cruz Biotechnology: anti-cyclin E1 (HE12), anti-cyclin E1 (C19), anti cyclin E1 (M20), anti-cyclin A2 (H432), anti-cyclin B1 (GNS1), anti-Ki67 (C20) and anti-p21 (C19). Other antibodies were anti-cyclin A2 (CY-A1, Sigma-Aldrich, Inc.), anti PCNA (167, Sigma-Aldrich, Inc.), anti-γ-tubulin (GTU-88; Sigma-Aldrich, Inc.), anti Aurora kinase B (Abcam), anti Keratin 1,10,11 (Ks 8.60; Abcam), anti Keratin 10 (RKSE60; Abcam), anti-keratin 1 (AF87; Covance), anti-γH2AX (Ser139; Millipore), anti-BrdU (B44; BD Biosciences), anti-phospho-histone H3 (P-H3; Upstate Biotechnology), anti-P-Rb (Ser780; Cell Signalling Technology), anti-P-Rb (Ser807-811; Cell Signalling Technology), anti HEC1 (9G3.23; Genetex) and Cdc14A (ZMD.231; Zymed). Secondary antibodies were coupled with fluorescein (FITC) or Texas Red (both from Jackson ImmunoResearch). For the study with confocal microscopy we stained the nuclei with propidium iodide to reveal nuclei and nucleoli (0,5 µg/ml) and we used a secondary antibody Cy5 (Jackson ImmunoResearch) for triple labelling. We obtained simple images (1,5 µm) and stacks of images with 1 µm steps. Confocal microscopy was performed with a laser scanning microscope (LSM 510; Carl Zeiss, Germany) by using excitation wavelengths of 488 nm (for FITC), 543 nm (for Texas Red) and 649 nm (for Cy5). Each channel was recorded independently and pseudocolor images were generated and superimposed.

### Isolating keratinocytes or nuclei from epidermis

The skin biopsies were washed in PBS and most fat removed and then cut into 0.5–1 cm long square pieces. The epidermis was detached of the skin by Dispase II at 50 mg/ml (Boehringer Mannheim) for 16 hours at 4°C. The epidermis was then peeled off with forceps from the underlying dermis as an intact sheet. The epidermal sheets were incubated with shaking in a trypsin solution (0,25% Tryp/EDTA 250 µM/PBS) at 37°C for 15 min for primary cultures and for a second 15 min for analyses. The proportion of isolated suprabasal, differentiating keratinocytes varied due to their strong cell attachments. Prolonging trypsin incubations resulted in cellular lysis. Keratinocytes were then filtered trough a 70 µm mesh and fixed for flow-cytometry (see below).

For isolating nuclei, epidermal sheets were cut in halves and fixed in cold 70% ethanol overnight at 4°C. Epidermal sheets were then incubated in pepsin solution (0,1 mg/25 mM HCl/PBS) for 40 minutes at 37°C. The suspension was washed three times in PBS, observed under the microscope and then filtered on a 40 µm mesh. Nuclei size and DNA content were then analysed by flow-cytometry (see below).

### Fluorescence *In Situ* Hybridisation

FISH was performed on human skin 20 µm-thick microsections *in situ* or on freshly isolated nuclei, to probe for specific chromosome centromeres or loci. For *in situ* studies, microsections were dried and fixed in 1/3 acetic acid/methanol, dried again and submerged in 0.25% trypsin for 2 min. They were then rinsed with absolute ethanol, dried and submerged in 4% formaldehyde/PBS for 15 min/0°C, washed in PBS and submerged in 0.01 HCl N for 5 min. They were then dehydrated in 75/85/100% ethanol for 1 min each and hybridised as below.

For FISH on isolated nuclei, keratinocytes were freshly isolated as above. Cells were centrifuged at 200×g for 10 minutes and the pellet was washed in PBS at 37°C, resuspended in 5 ml 75 mM KCL and incubated at 37°C for 20 min. After centrifugation for 10 min at 200×g, the pellet was slowly resuspended in 5 ml Carnoy4s fixative: methanol/acetic acid (3∶1,vol/vol). One drop of the cell suspension was spread on a microscope slide. The slides were air-dried. They were then dehydrated in increasing concentrations of ethanol, at 70, 80, 90 and 100% and air dried again.

Hybridisation on skin sections or isolated nuclei was performed as previously described [73]. DNA FISH chromosome probes were used for chromosome evaluation (VysisR). The probes were a spectrum orange directly labelled fluorescent DNA probe specific for AT rich alpha satellite DNA sequence at the centromeric region of each chromosome. The slides and probes were denatured for 5 min at 75°C in 70% formamide/2X SSC pH 7. Hybridization was carried out in a humid chamber for 30 min at 42°C. The slides were then washed in 4 x SSC at 75°C pH 7 for 3 s, then 2 x SSC 0.1% Igepal CA-630 (SigmaR) for 5 s at room temperature. The slides were then dried at 37°C in the dark, stained with DAPI and mounted in 10 µl anti-fade (Vectashield H 1000 VectorR). Slides were investigated under a LEICA fluorescence microscope and digital images were obtained using a computer ‘Cytovision Probe’ (Applied Imaging System). Almost 200 nuclei were examined for each individual slide on objectives with higher magnification (100 X) following the signal counting manufacturer’s guidelines. We enumerated the number of nuclei with 0, 1, 2, 3, or >4 signals and the nuclei with uncertain signals were not enumerated. Centromeric probes for chromosomes 8, 9, 18 and 20 were used. For specific locus probes for c-myc, Her 2 Neu and 9p were used.

### *In situ* DNA replication

The Method by Mills et al (2000) was adapted to skin sections [41]. Briefly, skin biopsies were rapidly snap-frozen, microsectioned and collected on glass-slides. Skin sections were then thawed and immediately were overlaid with 40 µl replication buffer, prepared with 30 µl of incubation buffer (0,25 M sucrose, 75 mM NaCl, 0,5 mM spermidin, 0,15 mM, spermin, 3% BSA) and 10 µl nucleotide mix (40 mM Khepes, 7 mM MgCl2, 0,1 mM dCTP, dATP, DGTP, rUTP, rCTP, rGTP, 60 mM ATP, 50 µM digoxigenin-UTP, 0,5 mM DTT, 80 mM creatin phosphate, 10 µg/ml phosphocreatin kinase). The PARP (poly (ADP-ribose)-polymerase) NU1025 inhibitor (Calbiochem) was added by routine to the replication buffer (200 µM). To test the specificity of the reaction, the DNA polymerase alpha and delta inhibitor Aphidicolin (Sigma-Aldrich, Inc.) was added in some cases to inhibit DNA replication (3 µg/ml).

Sections were incubated in a moist chamber for 15–45 min at 37°C. They were then washed in PBS and fixed/permeabilised in methanol (−20°C) for 10 min., washed and stained with fluorescein-labelled anti-digoxigenin (Roche) for 1 h at room temperature, washed again and mounted in Mowiol medium.

### *In toto* DNA synthesis

To analyse DNA synthesis in human epidermis *in toto*, about 2–3 mm pieces of epidermis were obtained as described above and incubated for three hours with 10 µM BrdU in complete FAD medium, with or without 10% serum. When larger epidermal pieces were used, BrdU was scarcely detected. Epidermal pieces were then washed with 250 µM EDTA in PBS, trypsinised twice for 20 minutes and cells resuspended, filtered and fixed in ice-cold 70% ethanol for BrdU/DNA staining and flow-cytometry analyses as described below. For *in situ* BrdU staining, epidermal pieces were snap-frozen in isopentane and labelled with anti BrdU antibody as above.

### DNA quantitation *in situ*

25 µm thick skin sections were fixed in cold methanol for 10 minutes and incubated with DAPI diluted in PBS (2 µg/ml) overnight at 4°C in a moist chamber to allow whole penetration and labelling of nuclear DNA. Stacks of images were obtained using confocal microscopy with 0,02 µm steps and 3D reconstructions were analysed with Imaris (Bitplane AG) or built and segmented with Volocity (Improvision Inc). Nuclei were segmented and those that had been cut and were incomplete disregarded. The sum of intensities and volume of each nuclei were calculated and compared. 3D reconstructions were performed over two different fields of two different sections per skin biopsy from three different body sites and individuals (foreskin, scalp, eyelid).

### Cell culture and treatments

Freshly isolated keratinocytes from foreskin of two different individuals KMC and KMD respectively (1 and 31 years old) were cultured in the presence of a mouse 3T3 fibroblasts (J2) feeder layer of in FAD medium as described previously [74]. Keratinocytes were treated with 0,5 µg/ml ICRF-193 (a generous gift from Jacques Piette) or 100 µg/ml Bleomycin (Bellon^R^) for 24 or 48 hours as indicated. After treatments, keratinocytes were trypsinised and fixed for flow-cytometry as described below.

### Flow-cytometry analyses

To analyse DNA synthesis or involucrin staining, keratinocytes were treated, fixed and stained as described elsewhere [15]. Keratinocytes were cultured in the presence of 10 µM BrdU for 2 hours before trypsin-harvest, harvested and fixed in 70% ethanol for keratin, DNA or BrdU staining. Fixed cell suspensions were treated with 2N HCl and for antibody staining, cells were incubated for 1 h with anti-keratin 1 (Babco) or anti-BrdU (B44, BD Biosciences) diluted and washed in 10% FCS/0,5% Tween 20/PBS. Then washed in PBS10%/FCS/0,5% Tween 20 and incubated with secondary antibody coupled to FITC diluted in the same solution for 1 h. Nuclear DNA was stained with propidium iodide (PI; 50 µg/ml) for cell cycle analyses. For involucrin staining, cells were fixed in 1% paraformaldehyde for 5 minutes, washed in PBS, permeabilized with saponin and stained, as previously described [15]. All antibody stainings were controlled with primary negative isotypes (mouse anti cd8 or normal rabbit serum). After staining, all cells were firmly resuspended and filtered trough a 70 µm mesh to minimize the presence of cell aggregates and then analysed by flow cytometry on a Beckton Dickinson FACScan. 10000 events were gated and acquired in list mode for every sample.

## Supporting Information

Data S1 **Additional results and references.**
(DOC)

Figure S1 **Expression of cell cycle regulators in epidermis.** Double staining of skin for **a**,**b**: cyclin E and keratin 1; **c–e**: cyclin A and keratin 1; **f**: cyclin A and keratin 5; **g**: cyclin A and involucrin (invol); **h**: cyclin B and involucrin; Colours as indicated. Nuclei were stained with Dapi, in blue. Note that Cyclin A patches are found mainly in the basal layer in some areas (c,d), or in peribasal layers in some others (c,e). Note also that cyclin A and cyclin B are bright in layers where the differentiation marker involucrin begins to be expressed (g,h). Dotted line indicates the basement membrane. SS: skin surface.
(TIF)

Figure S2 **Expression of cell cycle regulators of S phase in epidermis.** Double staining of skin for **a**: cyclin E and PCNA; **b–d**: PCNA and keratin 1; **e–f**: Ki67 and keratin 10; **g**: cyclin E and phospho‐Rb in ser780; **h** phospho‐Rb in ser780 and keratin 10, amplified area of the basal layer. **c**,**d**: amplified areas of suprabasal layers. Colours as indicated. Nuclei were stained with Dapi, in blue. PCNA is a DNA replication complex component. Ki67 is a cell cycle progression marker. PCNA was profusely expressed in the first suprabasal layers, with a sparser and punctuated pattern in more superficial layers (a–d), reflecting the multiple replication origins. Ki67 was expressed in most nuclei throughout epidermis (e–f). Arrow points at a ‘mushroom cell’, arrowheads at mitotic figures. Dotted line indicates the basement membrane, broken line a mushroom cell. SS: skin surface.
(TIF)

Figure S3 **DNA replication and cyclin A expression in the basal layers of epidermis.** In situ DNA synthesis (Digoxigenin incorporation; Dig, in green), cyclin A (red) and DNA (Dapi, in blue). Arrowheads indicate nuclei positive for cyclin A and for Dig, indicating that those cells are in S phase. Arrows indicate peribasal nuclei positive for cyclin A but not for Dig, suggesting they are in G2 phase. Asterisks indicate nuclei positive for DNA replication but not for cyclin A, suggesting they are endoreplicating.
(TIF)

Figure S4 **Expression of the DNA repair marker γH2AX in human skin sections.** **a–f**: expression of γH2AX in human foreskin (green, upper panels) and the corresponding DNA staining (blue; lower panels). **a**,**b**: Pre‐fixed skin was frozen, sectioned and re‐fixed 0 min after thawing (a), or 90 min after thawing (b). **c–f**: parallel unfixed skin was frozen, sectioned and fixed 0 min (c), 15 min (d), 60 min (e) or 90 min after thawing (f). In all cases fixation was in 4% formaldehyde. Note a faint background of γH2AX in the epidermal nuclei increasing from d to f. SS for skin surface. Dotted line for the basement membrane.
(TIF)

Figure S5 **DNA synthesis in skin in the presence of inhibitors of DNA repair or DNA replication.** Sections were stained for digoxigenin (Dig, green) and keratin 10 (K10, blue), as indicated and analysed by confocal microscopy. Nuclear DNA is stained in red (propidium iodide). Lower panels show the Dig labelling only. **a–d**: In situ DNA synthesis assays on skin sections in the absence of the nucleotide analogue Dig, as a control (a), or in the presence of Dig (b), Dig and DNA replication inhibitor Aphidicolin (c), or Dig and DNA repair inhibitor NU1025 (d). SS for skin surface. Broken line for the basement membrane.
(TIF)

Figure S6 **Flow‐cytometry analyses of keratinocytes isolated from epidermal sheets after *in toto* BrdU incubation. a–c**: corresponding to skin samples in Fig. 1a. **a**: total population; **b**: BrdU incorporation of the basal population according to light scattering (as in Fig. 1a; 87.7% of total); **c**: the suprabasal population by scattering (12.3% of the total); **d**: light scattering of the BrdU positive population in a. Numbers in histograms represent the proportion of cells within each gate with respect to the total population. Note that suprabasal BrdU cells are more frequent within total BrdU cells (24.0%) than suprabasal cells within total isolated cells (12.3%; Figure 1a).
(TIF)
