# Supplementary material for: A Mitosis Block Links Active Cell Cycle with Human Epidermal Differentiation and Results in Endoreplication
Source: PLoS One. 2010 Dec 20;5(12):e15701. doi: 10.1371/journal.pone.0015701 (PMC3004957; doi:10.1371/journal.pone.0015701)
Supplement: Data S1 — Additional results and references. (DOC) [file pone.0015701.s001.doc]

**Zanet et al SUPPORTING DATA 1**

**ADDITIONAL RESULTS**

**Artifactual DNA repair on skin sections**

We wished to investigate whether some of the DNA synthesis that we detected in epidermis was due to DNA reparative synthesis. To this aim, we made used of a well established DNA repair marker, the phosphorylation of histone H2AX in serine 139 (γH2AX; [1]). DNA repair shortly follows the expression of this marker after DNA damage. We first noted a faint background of γH2AX in the skin sections used for DNA replication in all nuclei of basal layers that was very dissimilar to the Dig staining (Figure S5a-d). This staining became brighter and generalised as sections were incubated longer at 37°C (Figure S5a-f). We pre-fixed parallel skin pieces before they were snap-frozen and stained the sections similarly for γH2AX, detecting no ‘natural’ manipulation-induced γH2AX in the skin (Figure S5). Therefore, a generalised artifactual DNA repair signal appears in the skin sections with time at 37°C after thawing, likely due to DNA damage during the process of freezing, thawing and/or sectioning. To further study the potential contribution of DNA repair to our DNA synthesis assays we added to the reaction either an inhibitor of the DNA polymerase α and δ that executes S-phase DNA replication (Aphidicolin from Sigma-Aldrich, Inc.; [2]; 3 μg/ml), or the DNA poly(ADP-ribose)-polymerase (PARP) inhibitor, that intervenes in DNA repair (NU-1025 Calbiochem; [3]). As shown in Figure S5, whereas the DNA replication inhibitor Aphidicolin almost abolished nucleotide incorporation in our assays, apart from a faint background, the DNA repair inhibitor did not. In fact, addition of NU-1025 improved the replication assays, likely because by blocking the generalised repair phenomenon, it allows all energy supply (ATP) and nucleotides for the DNA replication polymerase delta. We included the DNA repair inhibitor (200 µM) as a routine in all DNA replication assays and performed 30-45 min incubations.

**REFERENCES**

1. Fernandez-Capetillo O, Lee A, Nussenzweig M, Nussenzweig A (2004) H2AX: the histone guardian of the genome. DNA Repair (Amst) 3: 959-967.

2. Hardt N, Pedrali-Noy G, Focher F, Spadari S (1981) Aphidicolin does not inhibit DNA repair synthesis in ultraviolet-irradiated HeLa cells. A radioautographic study. Biochem J 199: 453-455.

3. Griffin RJ, Pemberton LC, Rhodes D, Bleasdale C, Bowman K, et al. (1995) Novel potent inhibitors of the DNA repair enzyme poly(ADP-ribose)polymerase (PARP). Anticancer Drug Des 10: 507-514.
