# Supplementary figures and images for: A Mitosis Block Links Active Cell Cycle with Human Epidermal Differentiation and Results in Endoreplication
Source: PLoS One. 2010 Dec 20;5(12):e15701. doi: 10.1371/journal.pone.0015701 (PMC3004957; doi:10.1371/journal.pone.0015701)

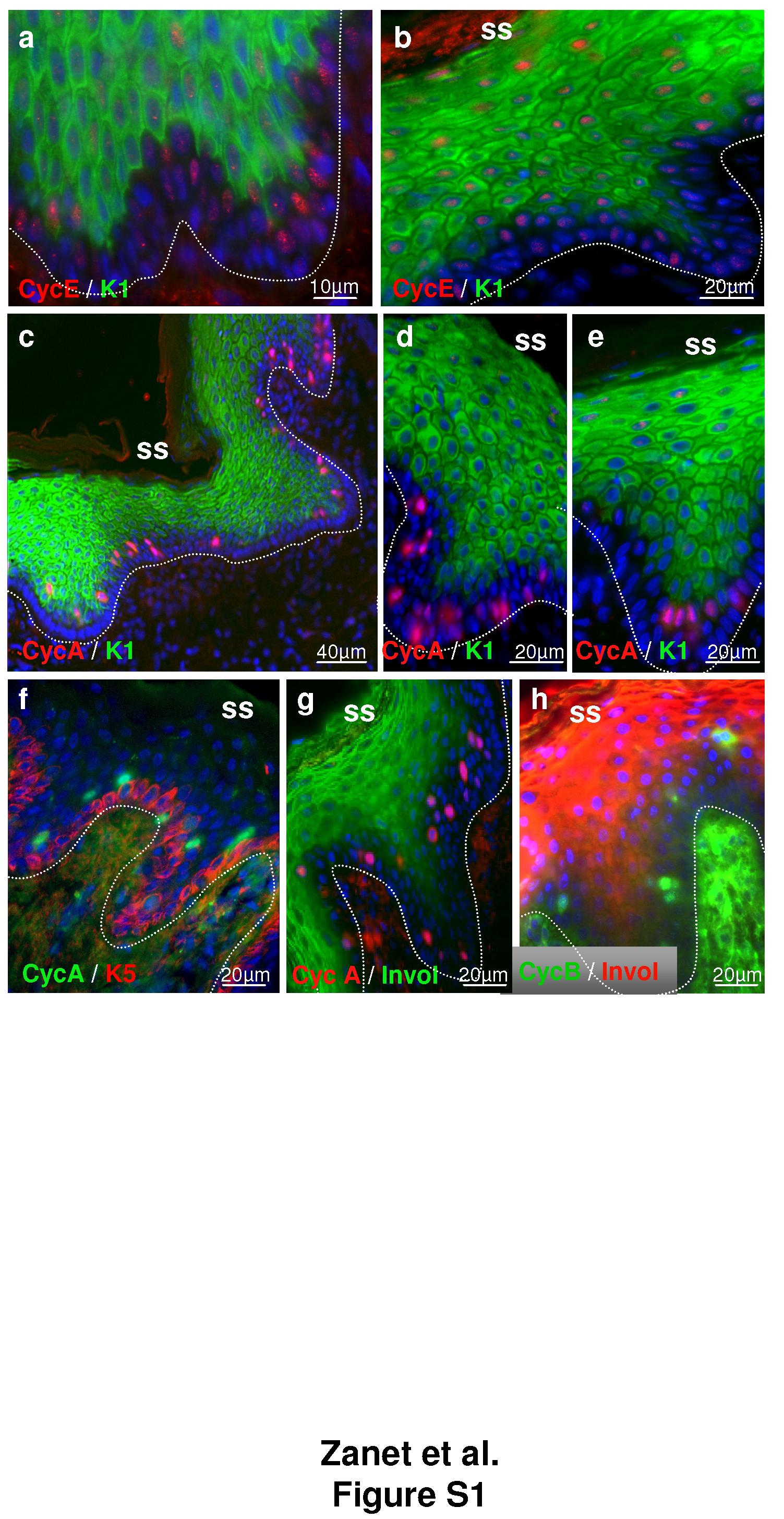

Supplement: Figure S1 — Expression of cell cycle regulators in epidermis. Double staining of skin for a,b: cyclin E and keratin 1; c–e: cyclin A and keratin 1; f: cyclin A and keratin 5; g: cyclin A and involucrin (invol); h: cyclin B and involucrin; Colours as indicated. Nuclei were stained with Dapi, in blue. Note that Cyclin A patches are found mainly in the basal layer in some areas (c,d), or in peribasal layers in some others (c,e). Note also that cyclin A and cyclin B are bright in layers where the differentiation marker involucrin begins to be expressed (g,h). Dotted line indicates the basement membrane. SS: skin surface. (TIF) [file pone.0015701.s002.tif]

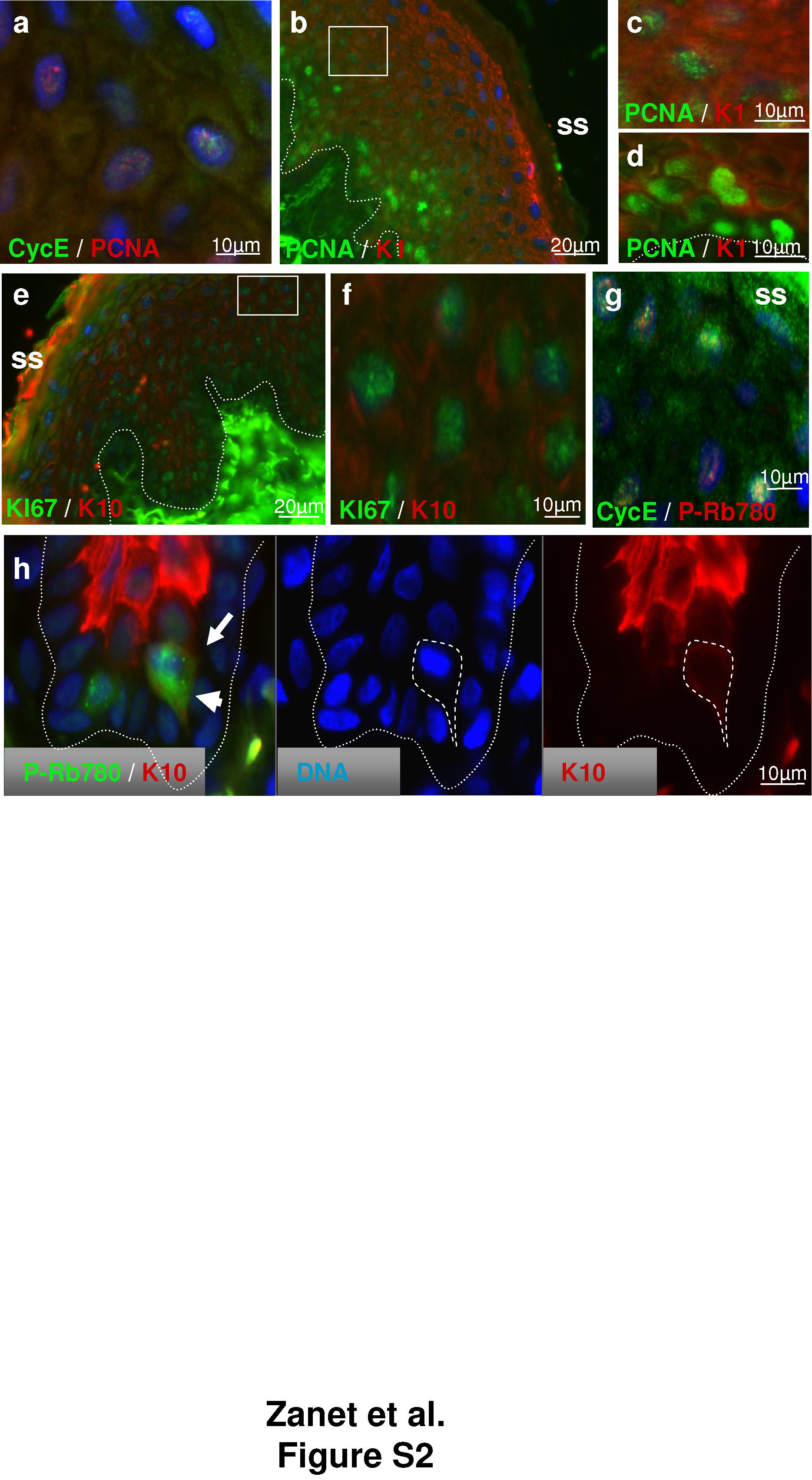

Supplement: Figure S2 — Expression of cell cycle regulators of S phase in epidermis. Double staining of skin for a: cyclin E and PCNA; b–d: PCNA and keratin 1; e–f: Ki67 and keratin 10; g: cyclin E and phospho‐Rb in ser780; h phospho‐Rb in ser780 and keratin 10, amplified area of the basal layer. c,d: amplified areas of suprabasal layers. Colours as indicated. Nuclei were stained with Dapi, in blue. PCNA is a DNA replication complex component. Ki67 is a cell cycle progression marker. PCNA was profusely expressed in the first suprabasal layers, with a sparser and punctuated pattern in more superficial layers (a–d), reflecting the multiple replication origins. Ki67 was expressed in most nuclei throughout epidermis (e–f). Arrow points at a ‘mushroom cell’, arrowheads at mitotic figures. Dotted line indicates the basement membrane, broken line a mushroom cell. SS: skin surface. (TIF) [file pone.0015701.s003.tif]

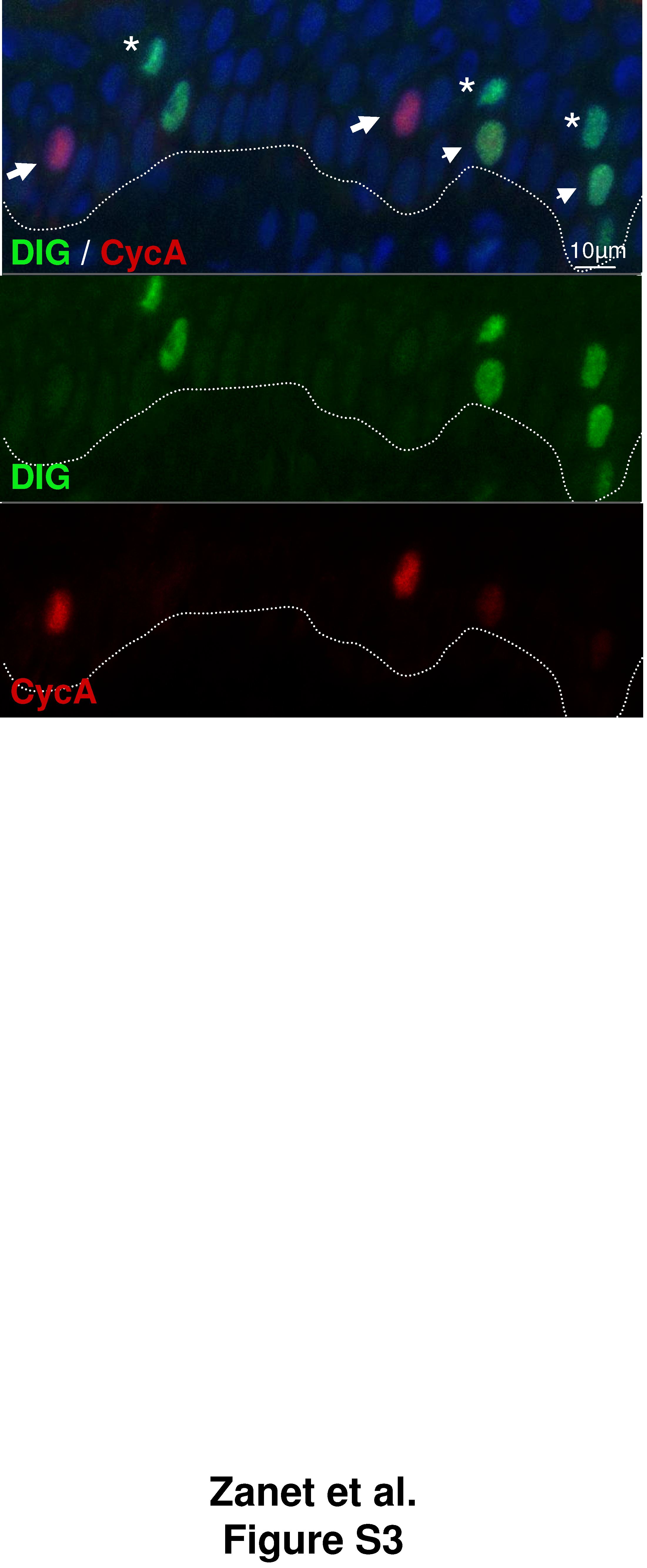

Supplement: Figure S3 — DNA replication and cyclin A expression in the basal layers of epidermis. In situ DNA synthesis (Digoxigenin incorporation; Dig, in green), cyclin A (red) and DNA (Dapi, in blue). Arrowheads indicate nuclei positive for cyclin A and for Dig, indicating that those cells are in S phase. Arrows indicate peribasal nuclei positive for cyclin A but not for Dig, suggesting they are in G2 phase. Asterisks indicate nuclei positive for DNA replication but not for cyclin A, suggesting they are endoreplicating. (TIF) [file pone.0015701.s004.tif]

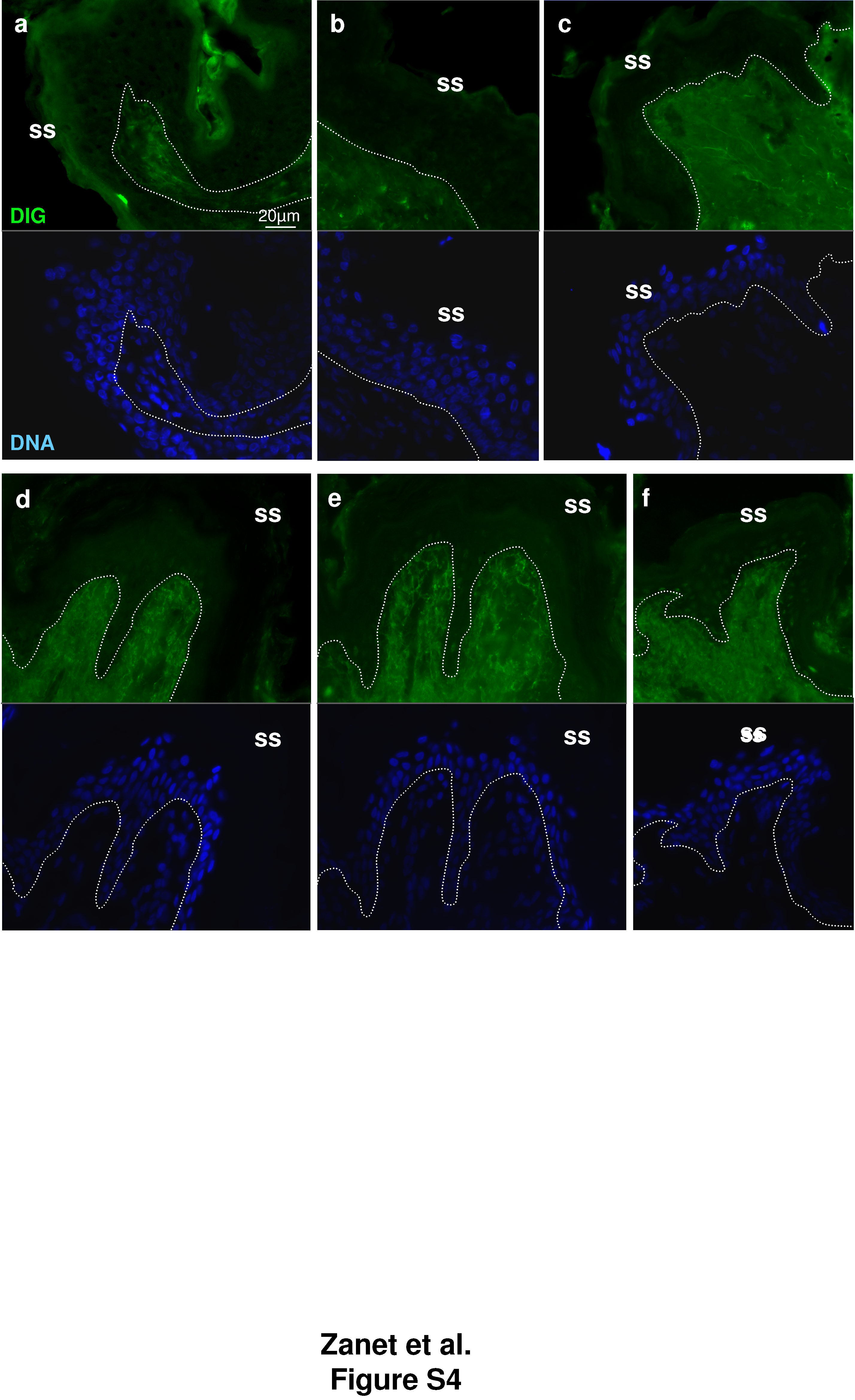

Supplement: Figure S4 — Expression of the DNA repair marker γH2AX in human skin sections. a–f: expression of γH2AX in human foreskin (green, upper panels) and the corresponding DNA staining (blue; lower panels). a,b: Pre‐fixed skin was frozen, sectioned and re‐fixed 0 min after thawing (a), or 90 min after thawing (b). c–f: parallel unfixed skin was frozen, sectioned and fixed 0 min (c), 15 min (d), 60 min (e) or 90 min after thawing (f). In all cases fixation was in 4% formaldehyde. Note a faint background of γH2AX in the epidermal nuclei increasing from d to f. SS for skin surface. Dotted line for the basement membrane. (TIF) [file pone.0015701.s005.tif]

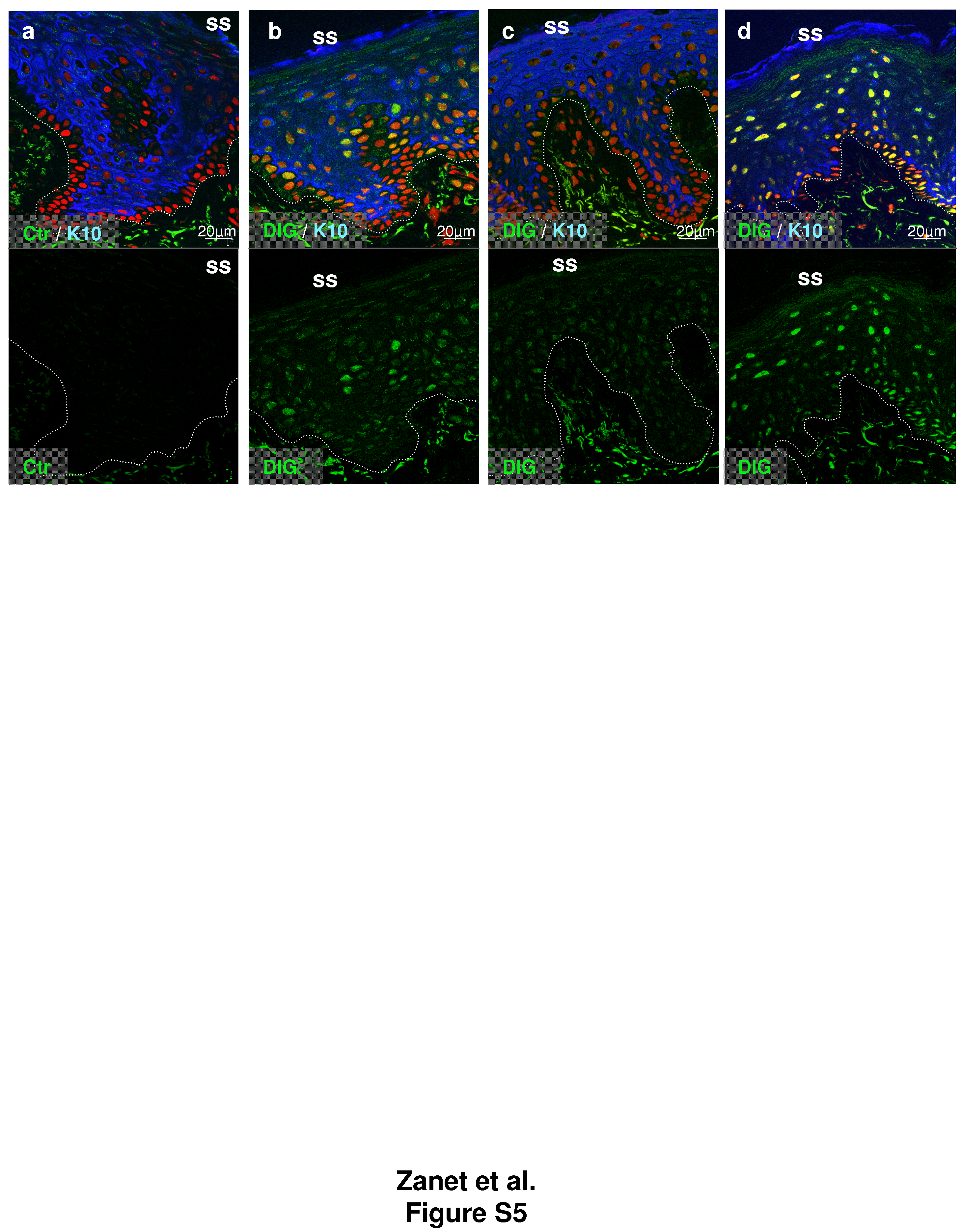

Supplement: Figure S5 — DNA synthesis in skin in the presence of inhibitors of DNA repair or DNA replication. Sections were stained for digoxigenin (Dig, green) and keratin 10 (K10, blue), as indicated and analysed by confocal microscopy. Nuclear DNA is stained in red (propidium iodide). Lower panels show the Dig labelling only. a–d: In situ DNA synthesis assays on skin sections in the absence of the nucleotide analogue Dig, as a control (a), or in the presence of Dig (b), Dig and DNA replication inhibitor Aphidicolin (c), or Dig and DNA repair inhibitor NU1025 (d). SS for skin surface. Broken line for the basement membrane. (TIF) [file pone.0015701.s006.tif]

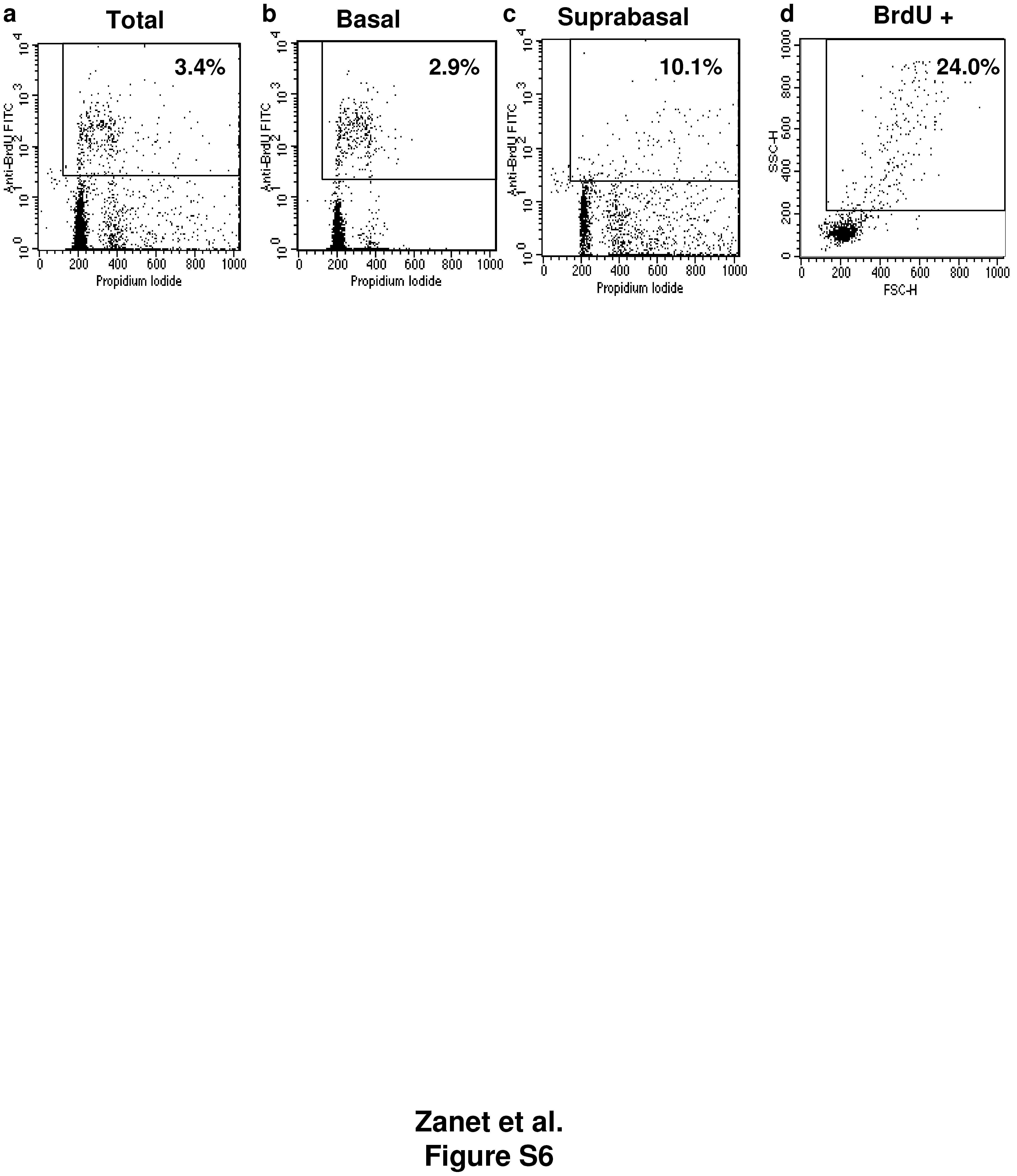

Supplement: Figure S6 — Flow‐cytometry analyses of keratinocytes isolated from epidermal sheets after in toto BrdU incubation. a–c: corresponding to skin samples in Fig. 1a. a: total population; b: BrdU incorporation of the basal population according to light scattering (as in Fig. 1a; 87.7% of total); c: the suprabasal population by scattering (12.3% of the total); d: light scattering of the BrdU positive population in a. Numbers in histograms represent the proportion of cells within each gate with respect to the total population. Note that suprabasal BrdU cells are more frequent within total BrdU cells (24.0%) than suprabasal cells within total isolated cells (12.3%; Figure 1a). (TIF) [file pone.0015701.s007.tif]
